# Genome-wide analysis of bHLH transcription factor family reveals their involvement in kernel development and biotic stress responses in Chinese chestnut

**DOI:** 10.3389/fpls.2025.1627760

**Published:** 2025-09-18

**Authors:** Rongchen Li, Liyun Huang, Xiaolu Huang, Jianming Liao, Xiaojuan Wei, Junji Li, Guangyu Zeng, Zhuogong Shi, Jingzheng Zhang, Zhiheng Zhao

**Affiliations:** ^1^ Guangxi Zhuang Autonomous Region Forestry Research Institute, Guangxi Key Laboratory of Characteristic Non-wood Forest Cultivation & Utilization, Guangxi Nanning, China; ^2^ Beijing Chemical Occupational Disease Prevention and Centrol Institute, Beijing, China; ^3^ College of Biological Sciences and Technology, National Engineering Laboratory for Tree Breeding, Beijing Forestry University, Beijing, China; ^4^ Research Center for Plateau Characteristic Agriculture in Northeast Yunnan, Zhaotong University, Zhaotong, China; ^5^ Hebei Normal University of Science & Technology, Qinhuangdao, China

**Keywords:** bHLH, biotic stress responses, evolutionary analysis, Chinese chestnut, transcription factor

## Abstract

**Introduction:**

The basic helix-loop-helix (bHLH) transcription factor family plays crucial roles in plant growth, development, and responses to environmental stresses. However, a systematic characterization of bHLH genes in Castanea mollissima has been lacking.

**Methods:**

We performed a genome-wide identification of bHLH genes in C. mollissima, followed by phylogenetic, structural, motif, chromosomal distribution, and cis-element analyses. Co-expression network analysis and transcriptomic profiling under Dryocosmus kuriphilus infestation were conducted, and representative genes were validated using qRT-PCR across seed developmental stages.

**Results:**

A total of 124 non-redundant bHLH genes (CmbHLHs) were identified and classified into eight subfamilies, consistent with conserved gene structures and motif compositions, particularly motif-1 and motif-2 of the canonical bHLH domain. Chromosomal mapping revealed non-random distribution and tandem duplication events. Promoter analysis indicated enrichment of cis-elements responsive to stress, hormone signaling, and developmental regulation. Co-expression analysis highlighted hub genes, including CmbHLH82 and CmbHLH57, with potential regulatory functions. Transcriptomic data showed that most CmbHLHs were downregulated under D. kuriphilus infestation, notably CmbHLH63 and CmbHLH51. qRT-PCR confirmed the dynamic expression of five selected genes during seed development.

**Discussion:**

These findings provide the first comprehensive overview of the bHLH gene family in Chinese chestnut. The results highlight candidate genes potentially involved in development and insect resistance, thereby laying a foundation for future functional and breeding studies.

## Introduction

Chinese chestnut (*Castanea mollissima*), belonging to the family Fagaceae, is native to China and Korea. It is rich in starch and dietary fiber, and, alongside dates and persimmons, is traditionally referred to as an “iron crop” and “woody grain” (Zhang S. et al., 2022). During the Middle Ages, *C. mollissima* served as a crucial food source in the northern hemisphere, acting as a grain substitute during periods of food scarcity (Nie et al., 2021; Zhou et al., 2021).This species exhibits strong tolerance to various abiotic stresses, including low temperatures, drought conditions, and soil depletion (Zhao et al., 2022; Zhu et al., 2022), as well as resistance to biotic stresses such as fungal pathogens and other diseases (Wang and Zhao, 2011; You et al., 2014). Owing to its high nutritional value, robust disease resistance, and adaptability to marginal environments, *C. mollissima* is recognized as an ecologically and economically important tree species. In recent years, significant progress in genome research has laid a solid foundation for the improvement of *C. mollissima* through breeding and targeted gene editing approaches (Xing et al., 2019; Nie et al., 2021).

Transcription factors are key regulatory molecules involved in virtually all aspects of plant growth and development, as well as responses to biotic and abiotic stresses, through selective binding to target gene promoters to regulate transcription (Strader et al., 2022; Zhang et al., 2023; Zou and Sun, 2023). Among them, the basic helix-loop-helix (bHLH) family plays a central role in processes such as photomorphogenesis, light signaling, secondary metabolism, and environmental stress responses (Yin et al., 2023; Zuo et al., 2023). bHLH proteins are also closely associated with hormone signaling pathways, including those of ABA, BRs, ethylene, gibberellin, and jasmonic acid (Du et al., 2023; Wang et al., 2022; Teng et al., 2023; Yuan et al., 2023). In addition, they contribute significantly to defense mechanisms against pests and drought stress (Kim et al., 2021; Kuang et al., 2023; Gao et al., 2023).

bHLH transcription factor family is one of the largest and most functionally diverse in plants, playing crucial roles in regulating growth, development, and responses to environmental stresses. Recent genome-wide studies across various species such as *Capsicum annuum* (Zhang H. F. et al., 2022), *Sorghum bicolor* (Fan et al., 2021a), *Aquilaria sinensis* (Sun et al., 2022), *Glycyrrhiza uralensis* (Ding et al., 2024), *Fagopyrum tataricum* (Sun et al., 2020), *Populus trichocarpa* (Zhang et al., 2024), Setaria italica (Fan et al., 2021b) and *Nicotiana tabacum* (Bano et al., 2021) have led to the identification and classification of numerous bHLH genes into distinct subfamilies based on conserved domains and phylogenetic relationships. Functional analyses have revealed that specific bHLH members are involved in pathways such as carotenoid and capsaicinoid biosynthesis, trichome and fruit development, and the modulation of abiotic stress responses including salinity, drought, and chilling (Fan et al., 2021a; Bano et al., 2021; Sun et al., 2022; Ding et al., 2024). For instance, CabHLH035 enhances salt tolerance by activating stress-related genes (Zhang H. F. et al., 2022), while PtbHLH35 and its homologs improve drought resistance through ROS scavenging and ABA signaling (Zhang et al., 2024). These findings highlight the multifaceted roles of bHLH transcription factors and lay a foundation for further investigation into their regulatory networks and potential applications in crop improvement.

With the recent completion and publication of the chestnut genome sequence (Li et al., 2024), a foundation has been established for functional genomic studies. However, bHLH transcription factor family in chestnut has not yet been systematically investigated, and the roles of *CmbHLHs* remain largely unknown. Additionally, *Dryocosmus kuriphilus* Yasumatsu, a widespread and destructive pest in chestnut-producing regions, causes severe damage to chestnut buds, yet the molecular mechanisms by which bHLH transcription factors respond to *D. kuriphilus* infestation are unclear. In this study, we performed a comprehensive identification and characterization of the CmbHLH gene family and conducted comparative analyses with bHLH genes from *Arabidopsis thaliana* (AtbHLH) and *Zea mays* (ZmbHLH). We further examined the expression profiles of *CmbHLH* during different stages of fruit development and under *D. kuriphilus* infestation. Several differentially expressed *CmbHLH* were validated by quantitative real-time PCR (qRT-PCR), and a putative protein-protein interaction network was constructed. These findings offer important insights into the potential roles of *CmbHLH* in chestnut development and stress response, laying the groundwork for future functional studies.

## Materials and methods

### Plant materials

The Chinese chestnut (*Castanea mollissima*) were collected from Yongren County, Chuxiong Prefecture, Yunnan Province, China. There are six varieties, namely ‘Yongfeng1’ (YF1), ‘Yongren zao’ (YRZ), ‘Yongfeng 2’ (YF2), ‘Yimen 2’ (YM2), ‘Yimen 1’ (YM1), and ‘Yanlong’ (YL), respectively. Developing spiny bur with nuts were collected at 10-day intervals, starting at 20 days until spiny bur matured into ripe fruit with cracked bulbs. Plant tissues were collected and immediately frozen in liquid nitrogen. There were three periods in total. The Chinese chestnuts collected in this study were cultivars, and Southwest Forestry University approved the experiment. The formal identification of the plant material was undertaken by Prof. Zhiheng Zhao. Our experimental research complied with local legislation, national and international guidelines.

### Identification and conserved motif analysis of *CmbHLHs*

To identify the bHLH genes in Chinese chestnut, the Hidden Markov Model (HMM) profile of the Pfam bHLH domain (PF00010) was downloaded from the pfam database (http://pfam.xfam.org/), and HMMER software (v3.1b2) (Potter et al., 2018) was performed to find the *CmbHLH* genes against in the Chinese chestnut genome (https://www.ncbi.nlm.nih.gov/search/all/?term=PRJNA912750). All *CmbHLH* candidates were further confirmed in the CD-search (NCBI database) (Marchler-Bauer et al., 2015) to verify the bHLH domain existing in each sequences, and any redundancy was manually removed. The conserved motifs of *CmbHLH* proteins were identified in Multipe EM for Motif Elicitation (MEME, http://meme-suite.org/).

### Multiple sequence alignment and phylogenetic tree construction

The *bHLH* sequences of *Arabidopsis thaliana* and *Zea mays* were downloaded from PlantTFDB (http://planttfdb.cbi.pku.edu.cn/). Using the ClustalX in MEGA X for multiple sequence alignment of constructed with 124 *CmbHLHs*, 225 *AthbHLHs, 323 TabHLHs*, and 308 *ZmbHLHs* (Meng et al., 2020), and Maximum Likelihood method was used to construct a phylogenetic tree with 1000 bootstrap sampling iterations in MEGA11 (Kumar et al., 2018). The amino acid sequences of bHLH proteins were aligned using MAFFT, and a phylogenetic tree was constructed using MEGA X based on the JTT substitution model. The resulting tree was visualized and annotated using iTOL (https://itol.embl.de/).

### Analysis of cis-acting elements in the promoter regions of CmbHLH

The 2,000 bp upstream promoter sequences of 124 chestnut CmbHLH transcription factor genes were extracted using TBtools. Cis-acting elements were identified via the PlantCARE database (http://bioinformatics.psb.ugent.be/webtools/plantcare/html/). The elements were subsequently filtered and grouped based on similar regulatory functions. A heatmap was generated using TBtools, and the number of cis-acting elements in each functional category was statistically analyzed.

### Gene structure analysis

The exon–intron structures of the identified genes were analyzed using the Gene Structure Display Server (GSDS) v2.0 (http://gsds.gao-lab.org/). The coding sequences (CDS) and corresponding genomic sequences were aligned to determine exon and intron positions. The resulting gene structure models were visualized to examine the structural diversity among gene family members.

### Expression analysis

In this study, the dynamic changes of *CmbHLH* genes in six chestnuts varieties were analyzed during fruit development and gall-information. The RNA-seq data of six chestnuts varieties flower and fruit development came from our previous study (PRJNA574282). And the RNA-seq data of chestnut in response to Dryocosmus kuriphilus Yasumatsu were downloaded from NCBI (PRJNA512447) (Zhu et al., 2019). Clean data were obtained from raw sequencing reads using fastp v0.20.1 (Chen et al., 2018), which removes adapter sequences and trims reads containing poly-N and low-quality bases (reads with more than 50% of bases having a Phred quality score ≤20). Clean reads from all samples were mapped to the *C. mollissima* genome (https://www.hardwoodgenomics.org/Genome-assembly/1962958) by using HISAT2 software for further analysis (Kim et al., 2019). Then all transcripts were quantified by Salmon 1.4 (Patro et al., 2017). Gene expression profiling was performed using FPKM (Fragments Per Kilobase of transcript per Million mapped reads) values. The raw FPKM values generated by Salmon v1.4 were used directly for expression pattern analysis without further normalization, as FPKM inherently accounts for transcript length and sequencing depth. Gene co-expression networks were constructed based on FPKM expression values using the WGCNA (Weighted Gene Co-expression Network Analysis) R package (v1.71). An appropriate soft-thresholding power was selected based on the scale-free topology criterion. Modules of co-expressed genes were identified using hierarchical clustering and dynamic tree cutting. The module–trait relationships were calculated to identify modules associated with specific conditions or developmental stages. The resulting networks were visualized using Cytoscape (v3.9.1).

### Protein-protein interaction network prediction

124 ZjbHLH protein sequences were used as queries, and protein-protein interactions were predicted by the STRING website (https://string-db.org/). The orthologs of Arabidopsis thaliana and Zea mays were selected as references. Finally, an interaction network among *CmbHLHs* was constructed in this study.

### qRT-PCR analysis

Total RNA was extracted using a plant RNA extraction kit (Vazyme Biotech) according to the manufacturer’s instructions. The construction of a cDNA library was performed by reverse transcribing 1 mg RNA samples using the 5×HiScript^®^ Reverse Transcriptase (vazymes) and 4×gDNA (vazymes) kits, following the manufacturer’s protocol. The expression of representative genes was analyzed by qRT-PCR in at least four biological replicates. The primer sequence is in **Supplementary Table S1**.

Actin was chosen as the reference genes. Relative expression levels of qRT-PCR data were calculated using the 2^-ΔΔCt method, and data are presented as mean ± standard deviation (SD). Statistical significance was tested using one-way ANOVA, corrected for multiple comparisons using Tukey’s HSD. A P value < 0.05 was considered statistically significant. All statistical analyses were performed using GraphPad Prism software.

## Results

### Genome-wide identification of *bHLH* genes from *Castanea mollissima*

A total of 124 non-redundant bHLH proteins were identified in the *Castanea mollissima* genome based on the Hidden Markov Model search and confirmed that them have typical *bHLH* domain. These 124 putative bHLH protein were renamed as shown in **Supplementary Table S1**. The encoded bHLH protein ranged from 86 (*CmbHLH94*) to 1241 (*CmbHLH19*) amino acids (aa), with Open Reading Frame (ORF) ranging from 258 bp to 3723bp in length. Based on the different physical and chemical properties of *CmbHLH*, it is suggested that they are likely have multiple functions.

### Phylogenetic analysis of *CmbHLH* protein

**Figure 1:**
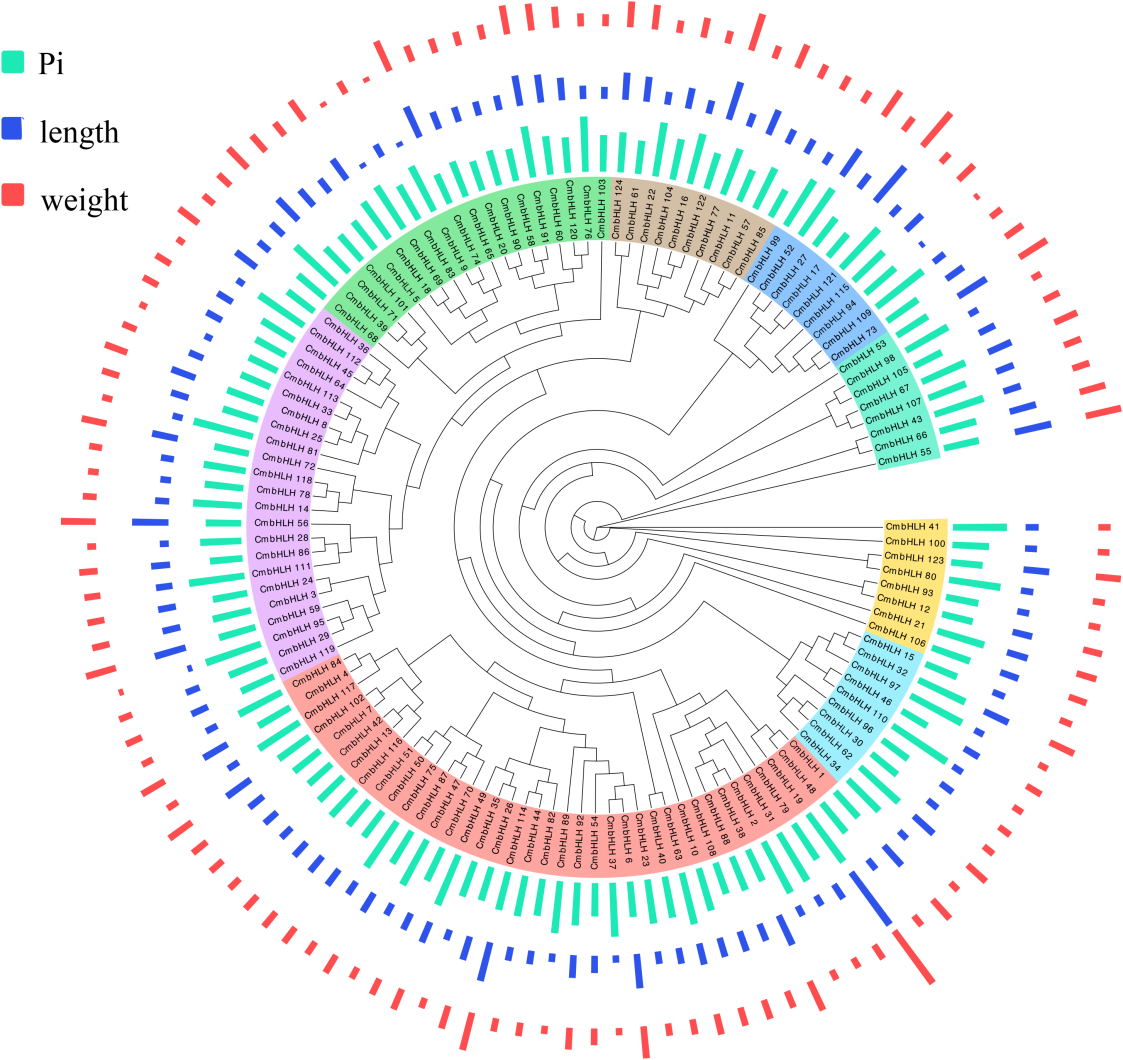
The phylogeny, grouping and basic characteristics of *CmbHLH*. The unrooted Max-likelihood phylogenetic tree was constructed with MEGA 11 software using the full-length amino acid sequences of 124 *CmbHLH* proteins. The 8 subfamilies are marked with different colors. In the outer circle of the phylogenetic tree, corresponding from the inside to the outside The PI, protein length and molecular weight of *CmbHLH*.

**Figure 2:**
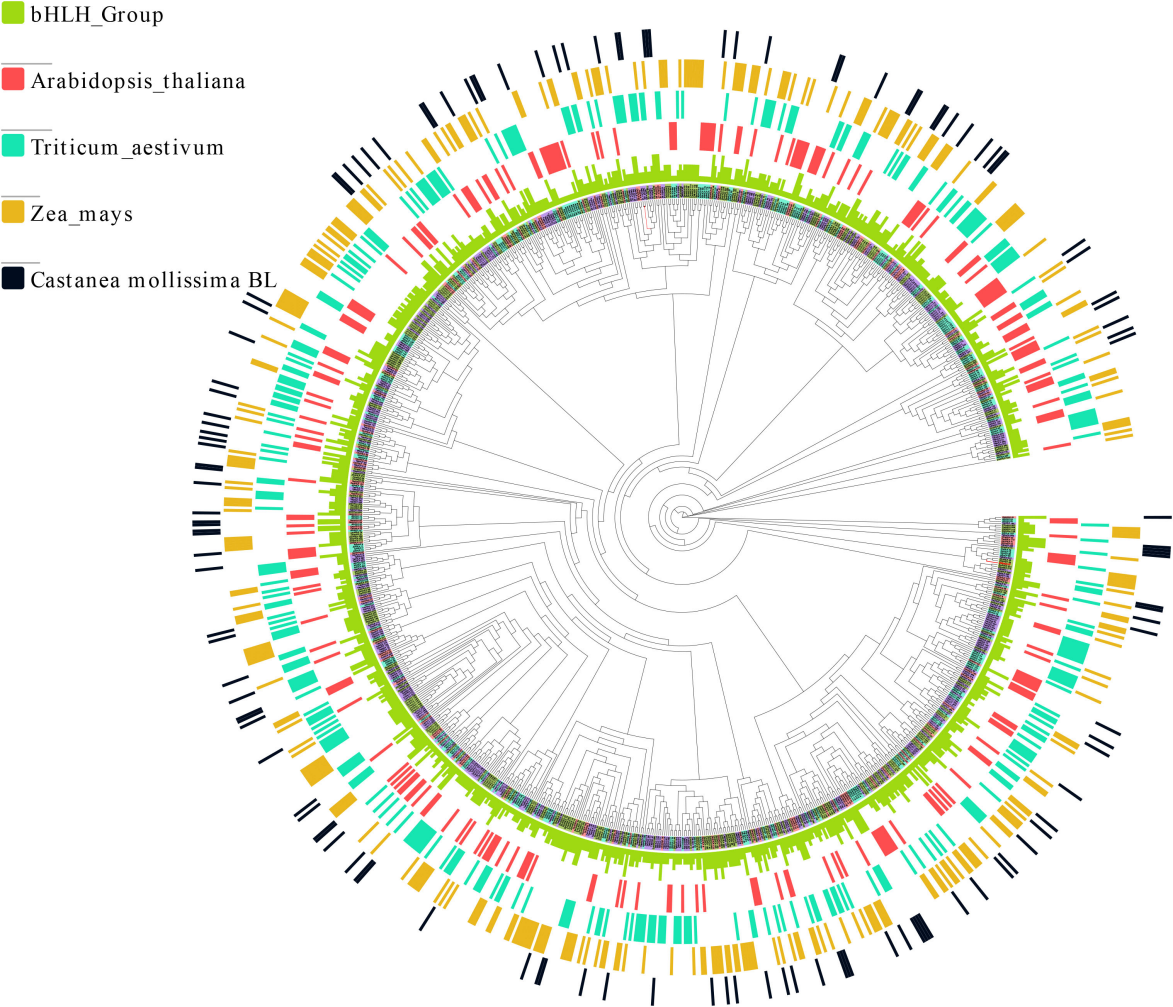
Phylogenetic tree of Castanea mollissima BL, Triticum_aestivum, Zea_mays, and Arabidopsis_thaliana bHLH proteins. The unrooted Max-likelihood phylogenetic tree was constructed with MEGA 11 software using the full-length amino acid sequences of 981 bHLH proteins. The different colors of the sequence names represent the bHLH sequences of different species. In order to better distinguish the evolution patterns of bHLH among different species, the bar graphs in the outer circle of the phylogenetic tree represent the bHLH genes of different species.

Based on the maximum likelihood (ML) phylogenetic tree in *CmbHLH* proteins, 124 *CmbHLH* were divided into eight subgroups and were named as group I to VIII. Among them, *CmbHLH*_I and *CmbHLH*_VIII had the fewest members, with seven and eight genes respectively. Within each subgroup, there was a high level of concordance in gene length and physicochemical properties among members of the same gene family, implying their potential co-localization on either the same or adjacent chromosomal loci (**Figure 1**). The phylogenetic analysis suggests that *CmbHLH*_I and *CmbHLH*_VIII potentially represent the most ancient subgroups in *C. mollissima*, while the gene family *CmbHLH*_II could be considered more recent (**Figure 1**). Additionally, the *CmbHLH*_VI subgroup exhibited the highest degree of differentiation and expansion, suggesting a lower level of conservation compared to the other subgroups, demonstrating that *CmbHLH*_VI is a dynamically evolving bHLH subgroup in *C. mollissima* (**Figure 1**). In order to futher explore the function and classification of *CmbHLH* protein, the ML phylogenetic tree was constructed with 124 *CmbHLHs*, 225 *AthbHLHs* from *Arabidopsis thaliana, 323 TabHLHs from T*riticum_aestivum, and 308 *ZmbHLHs* from *Zea mays* (**Figure 2**). The *CmbHLH* proteins were divided into 8 subgroups, named from I to VIII, according to the classification of *Arabidopsis thaliana* and *Zea mays*. The phylogenetic tree showed that the majority of orthologous *bHLH* genes are closely related in all three species, which indicates that most *bHLHs* arose prior to species divergence. *CmbHLHs*, which are relatively distant among species and relatively closely related within species, may have originated from the duplication event of paralogous genes.

### The chromosomal location and gene structure of *CmbHLHs*

**Figure 3:**
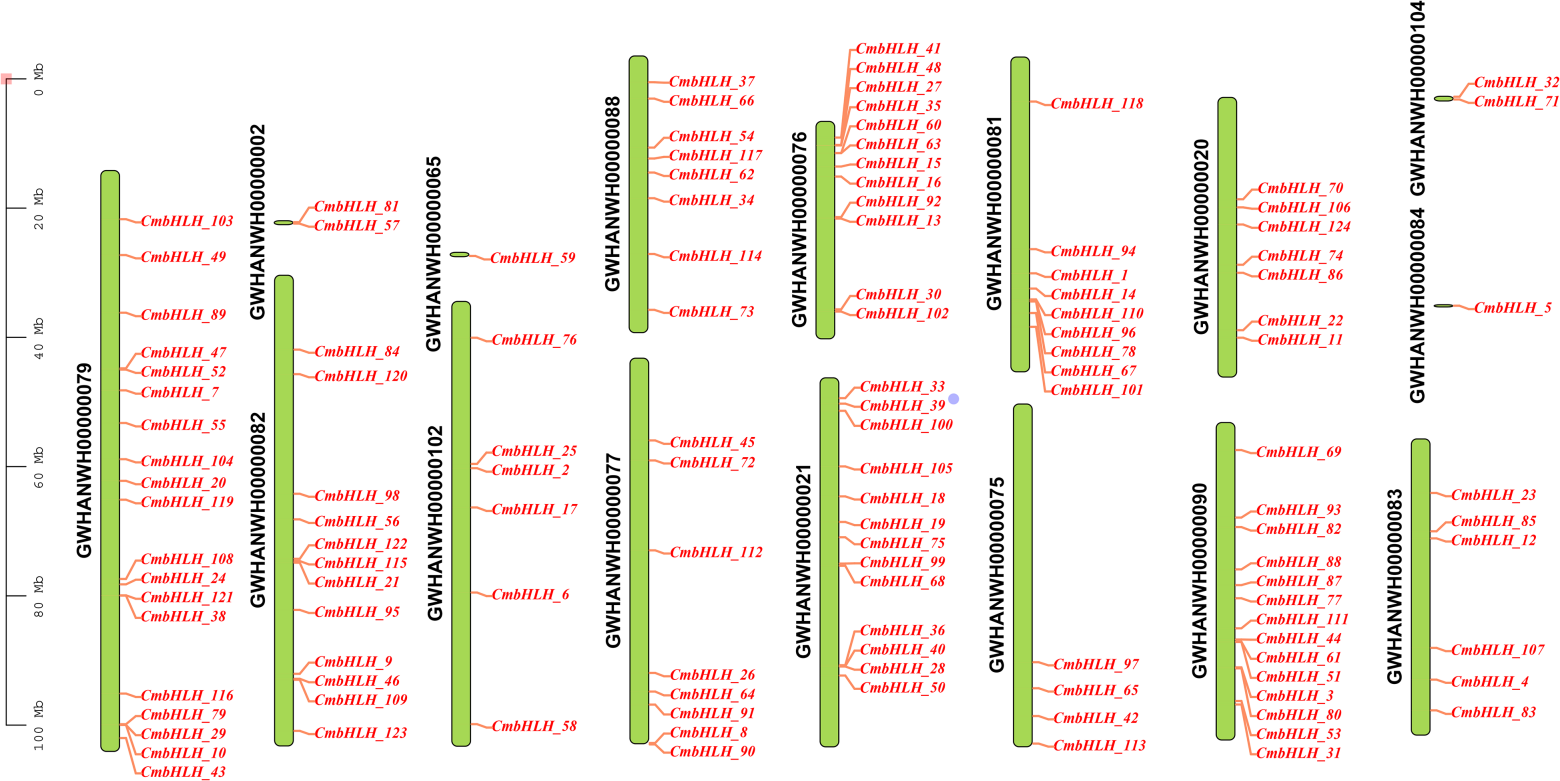
Chromosomal distribution of *CmbHLHs*. The coordinates on the left show the length of different chromosomes and contigs. 124 *CmbHLHs* are distributed on 12 different chromosomes and 4 contigs.

**Figure 4:**
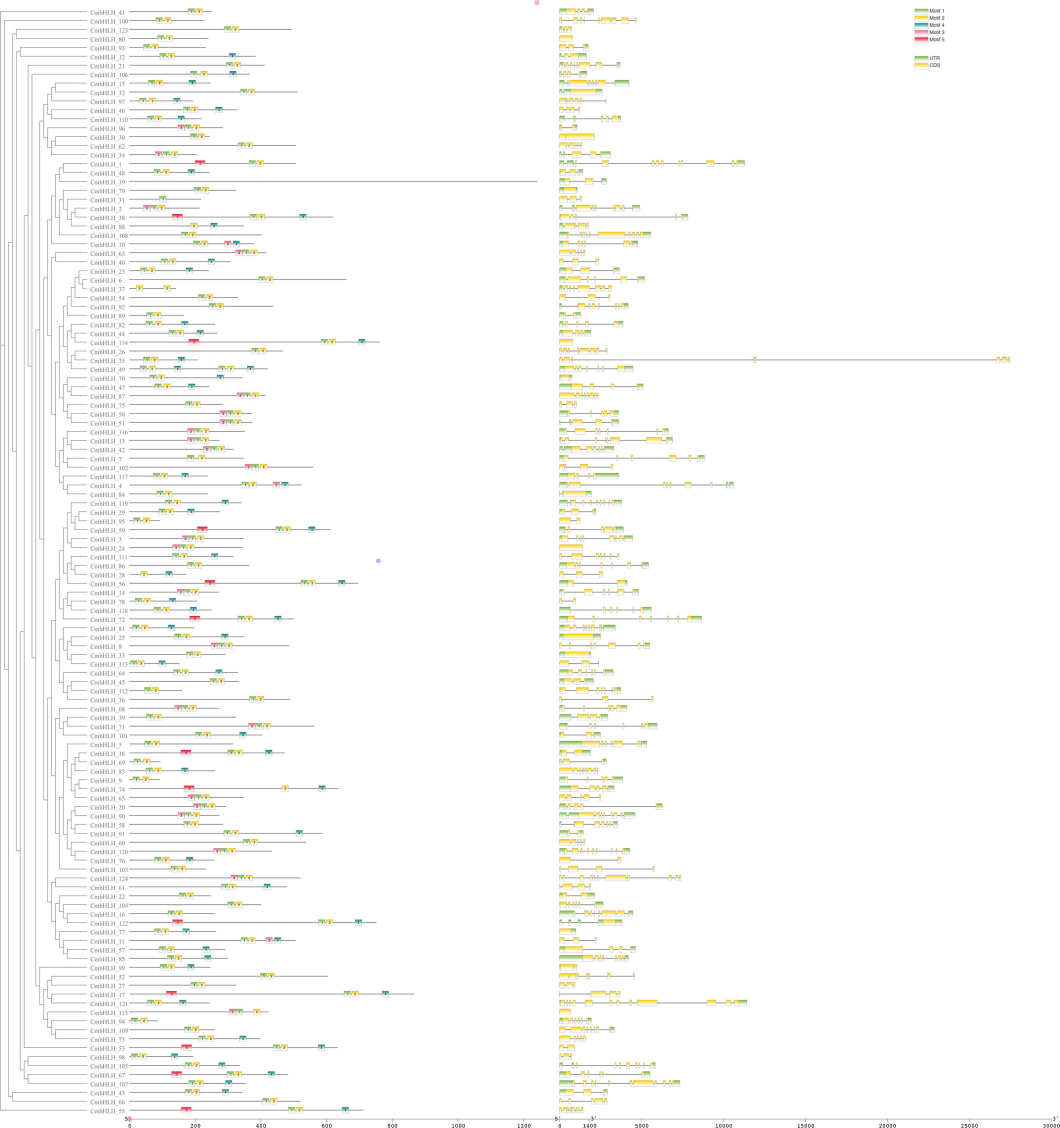
Gene structures of Castanea mollissima BL. basic/helix–loop–helix (*CmbHLH*) genes and phylogenetic relationships, conserved motifs of *CmbHLH* proteins. The structure diagram behind the phylogenetic tree is the distribution diagram of Conserved motifs. Different colors in Conserved motifs represent different motifs. The figure shows the position distribution of conserved motifs on all *CmbHLHs*. The final structure diagram is the gene structure diagram of all *CmbHLH*, where green represents the upstream and downstream non-coding regions of the gene, yellow represents the exon region, and the colorless line represents the intron region.

The 124 *CmbHLHs* were mapped to 12 different chromosomes and 4 different contigs (**Figure 3**). Among them, the *CmbHLHs* genes located on chromosomes GWHANWH00000079 and GWHANWH00000090 were the most, with 19 and 14 bHLHs genes, respectively (**Figure 3**). Interestingly, some genes are tightly clustered together. Interestingly, some of the genes were tightly packed into clusters to form tandem repeats (*CmbHLH68* and *CmbHLH99*; *CmbHLH47* and *CmbHLH52*), most of these genes belong to the same subfamily. A previous study analyzing repetitive events in rice and Arabidopsis indicated that some bHLH subfamily members are most likely derived from repetitive events. Also, analysis of the gene structure of all *CmbHLHs* shows that the bHLHs gene structure of *C. mollissima* contained 1–5 motifs, indicating that the gene family was highly conserved in terms of gene structure (**Figure 4**). it showed that motif-1 and motif-2 ware typical domains within the bHLH gene family in *C. mollissimat*. The subfamily of *CmbHLHs* VIII, with the exception of *CmbHLH*_106, exclusively contains motif-1 and motif-2, providing further evidence for the high conservation of *CmbHLHs* VIII within the *C. mollissimat* bHLH gene family (**Figure 4**).

### Multiple sequence alignment and conserved motifs in *CmbHLHs*

**Figure 5:**
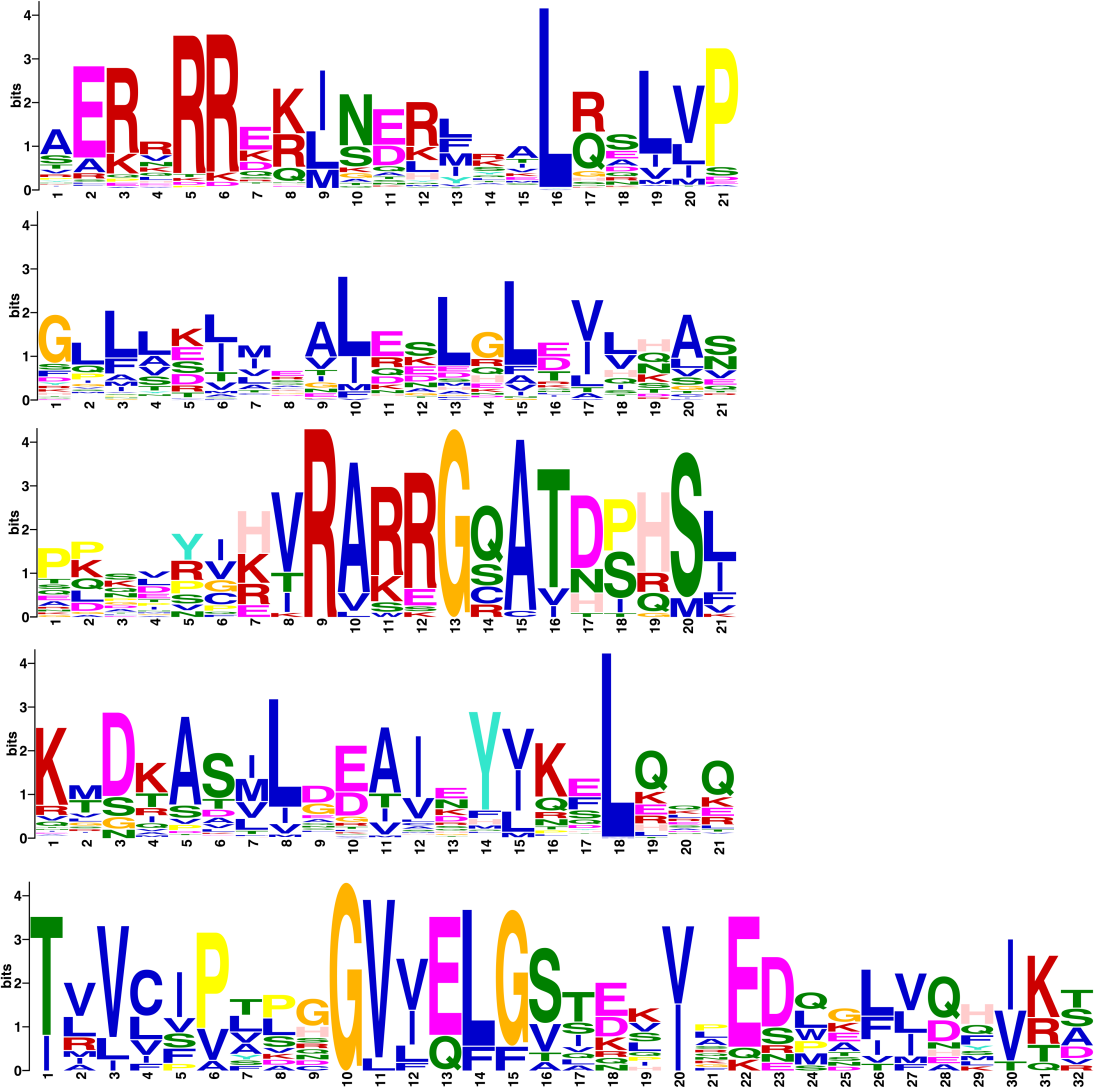
Sequence logo diagram of 124 *CmbHLH* Conserved motifs. The figure shows the sequence logos of 5 Conserved motifs, among which motif-1, motif-2, and motif-3 respectively present the typical conserved domains of the bHLH gene family. Motif-1,2,3 correspond to the helix, loop, and helix structures in the conserved domain of bHLH, respectively.

Multiple sequence alignment and MEME analysis showed that all *CmbHLHs* genes share the same conserved domains motif-1 and motif-2 (**Figure 4**). In addition, 18 CmHLHs genes such as *CmbHLH63, CmbHLH60, CmbHLH74, CmbHLH128, CmbHLH44, CmbHLH80, CmbHLH79, CmBEE3, CmbHLH59a, CmbHLH59b, CmbHLH122, CmbHLH62a, CmbHLH130a, CmbHLH137, CmbHLH62b, CmbHLH130b, CmbHLH31* and *CmbHLH49* contain motif- 3 (**Figure 4**). The structure and sequence of the CmHLHs gene family are highly conserved, with motif-1 and motif-2 forming the complete bHLH domain in the *CmbHLHs* genes family. Five residues (motif-1: Ala-1, Glu-2, Arg-3, Arg-5, Arg-6), five residues (motif-1: Arg-12, Leu-16, Leu-19, Val-20, Pro-21), two residues (motif-2: Gly-1, Leu-3), and six residues (motif-2: Ala-9, Leu-10, Leu-13, Leu -15, Val-17, Ala-20) constitute the basic region of the bHLH domain, the first helical region, the loop region and the second helical region (**Figure 5**), respectively. Motif-3,4,5 are also highly conserved domains. Of the 21 amino acid sequence, 15 residues are more than 50% amino acid conserved. Seven of these residues (motif-3: Arg-9, Ala-10, Arg-12, Gly-13, Ala-15, Thr-16, Ser-20) are 100% amino acid conserved suggesting that the 18 *CmbHLHs* containing motif-3 may be a very important and functionally similar transcription factor in chestnut.

### Analysis of cis-acting elements of CmbHLHs

Cis-acting element analysis was performed on the bHLH gene family in chestnut to explore the potential functions of CmbHLH transcription factors. All 124 CmbHLH transcription factors contained cis-elements in their promoter regions related to responses to biotic and abiotic stresses, growth and development, and hormone signaling (**Supplementary Figure 1**). The promoter regions of the CmbHLH transcription factors harbored multiple anaerobic induction elements (ARE), light-responsive elements, and drought-responsive elements (MBS). Notably, CmbHLH_77, CmbHLH_15, and CmbHLH_83 contained a relatively high number of ARE elements, while 60 CmbHLH transcription factors possessed a larger number of MBS elements.

Among common hormone-responsive elements, CmbHLH_35 contained 10 methyl jasmonate-responsive elements (TGACG-motif), and both CmbHLH_4 and CmbHLH_55 contained 8 TGACG-motifs. Additionally, 45 CmbHLH transcription factors harbored multiple salicylic acid-responsive elements (TCA-element), while CmbHLH_35, CmbHLH_46, and CmbHLH_54 possessed multiple abscisic acid-responsive elements. In terms of growth and development, 45 CmbHLH family members contained growth-regulating G-box elements, and 9 CmTrihelix family members included meristem expression-related CAT-box elements. These findings suggest that CmTrihelix transcription factors play crucial roles in plant responses to abiotic stress, hormone signal transduction, and growth and developmental regulation.

### *CmbHLHs* protein-protein interaction network prediction

**Figure 6:**
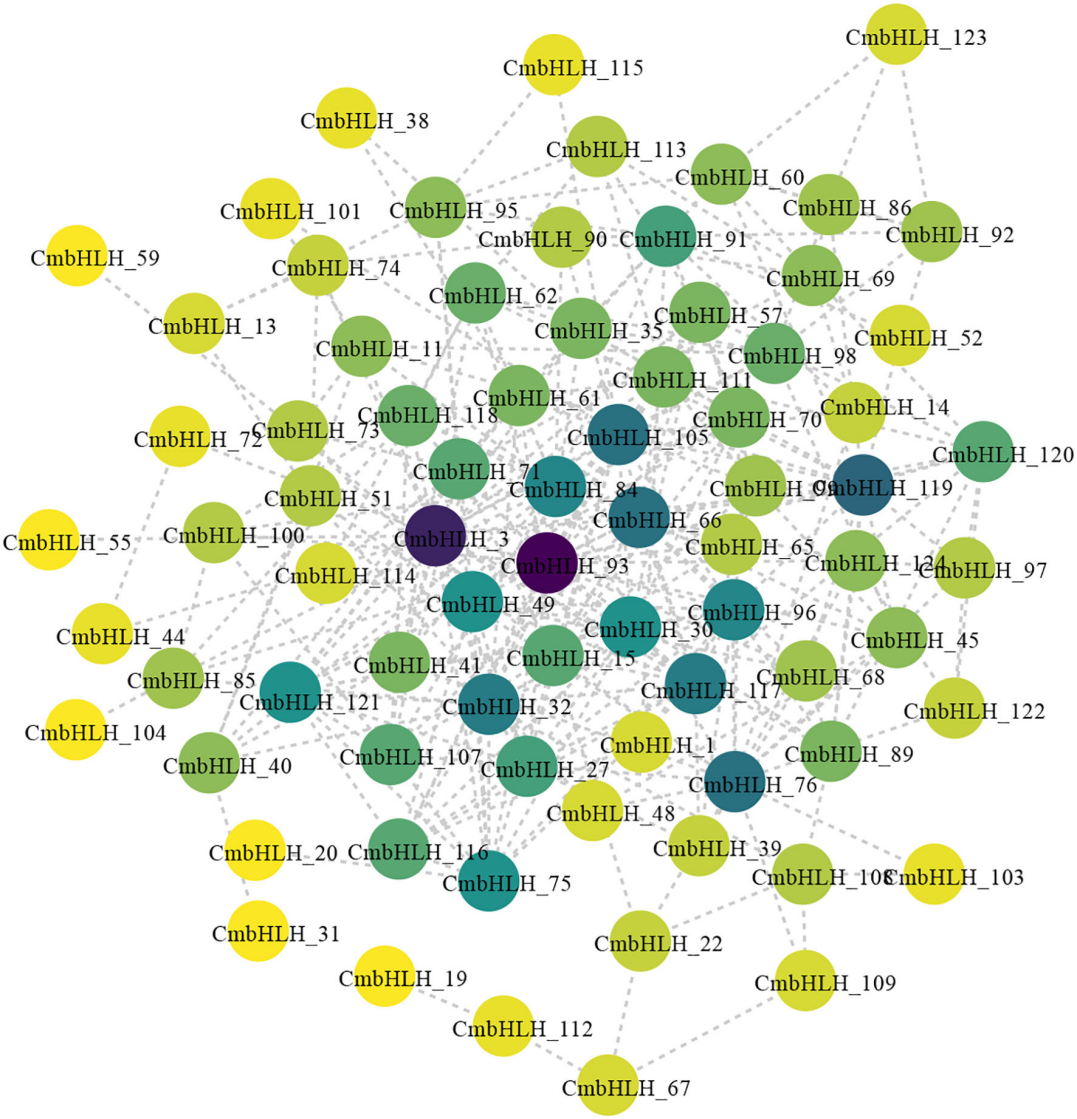
PPI network diagram of all *CmbHLH* genes. The 124 *CmbHLH* amino acid sequences were submitted to the string database, and the PPI network and functions among the 124 *CmbHLHs* were analyzed by comparing the homologous genes in Arabidopsis with default parameters.

Based on the orthologs in Arabidopsis and maize, STRING predicted interactions between *CmbHLHs* genes. Several important interactions are predicted in **Figure 6**, where *100282922* (*CmbHLH122*) may be involved in cuticle development, regulation of stomatal movement, photoperiodism and flowering. And *bHLH29* (*CmbHLH29*) may participate in the regulation of iron ion transport and the response to iron ion. While genes such as *AT2G40200* (*CmbHLH57*), *SPCH* (*CmbHLH98*), *bHLH104* (*CmbHLH104*), and *AT2G14760* (*CmbHLH84*) are grouped into one module on the PPI network diagram. And it is speculated that the function of these genes may be related to stoma formation and regulation of osmotic pressure. The concentration of genes such as AT1G31050 (*CmbHLH*111), *AT5G50915* (*CmbHLH137*), and *BPEp*, and *UNE12* in one module may indicate that these transcription factors may be related to petal development and gamete formation. These results further proved the functional diversity of *CmbHLHs* genes. The predicted network provides some useful clues for functional studies, but further experimental evidences is needed.

### *CmbHLHs* involved in flower and fruit development in different chestnut varieties

**Figure 7:**
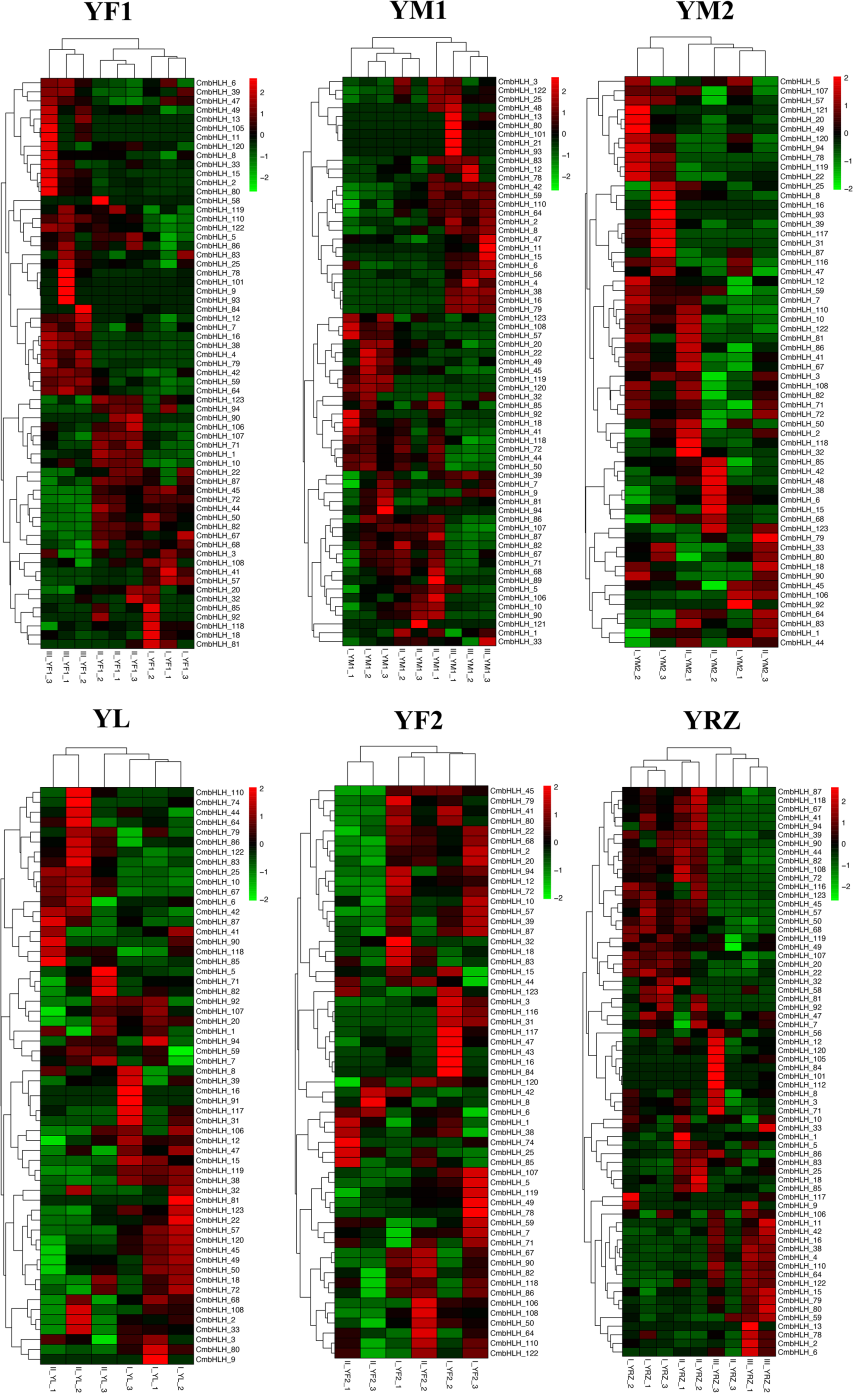
Heat maps of the differentially expressed *CmbHLH* genes in the different flowering stages of 6 chestnut varieties (YF1, YM1, YM2, YL, YF2, YZ). Among them, YF1, YM1, YRZ showed differential expression in three flowering stages, and YM2, YL, YF2 only showed differential expression in two flowering stages. In general, *CmbHLH* showed stage-specific expression in different stages of chestnut flowering. Among them, the expression of *CmbHLH* in YF1, YM1, and YRZ showed similarity. There were a large number of *CmbHLH*-specific high expressions in stage III, and only a few *CmbHLH*-specific high expressions in stage I and II. YM2 and YL showed different expression patterns. *CmbHLH* showed more specific high expression in stage I, but less in stage II.

Previous studies have shown that genes of bHLH transcription factor family genes are widely involved in plant development, fruit ripening, and abiotic stresss (Atchley et al., 1999; Massari and Murre, 2000; Carretero-Paulet et al., 2010; Zhang et al., 2018). To identify *CmbHLHs* genes that have a significant role in chestnut flower and fruit development, transcriptome data of 6 chestnut varieties at different stages of fruit development were analyzed. The results showed that 84 out of the 124 *CmbHLHs* genes were differentially expressed during chestnut fruit development (log2|FC|>=1, p<0.05) (**Figure 7**). Among them, *CmbHLH*4, 7, 16, 38, 59, 64 and other genes were mainly expressed in the early and middle stages of chestnut fruit development and most of these genes belonged to subgroup V and VI. While genes such as *CmbHLHs* 11, 13, 15, 25, 93, 101, 122 were mainly expressed in the late stage of chestnut fruit development. The trends of these genes in different varieties were basically the same in all 6 varieties. In general, the expression of *CmbHLHs* exhibited stage-specific patterns during the maturation process of chestnut kernels. Among them, the expression of *CmbHLHs* in YF1, YM1, and YRZ showed similarity. There were a large number of *CmbHLH*-specific high expressions in stage III, and only a few *CmbHLH*-specific high expressions in stage I and II. YM2 and YL showed different expression patterns. *CmbHLHs* showed more specific high expression in stage I, but less in stage II. The results show that these genes are essential transcription factors for fruit development in chestnut, and that they play an important role in fruit development.

**Figure 8:**
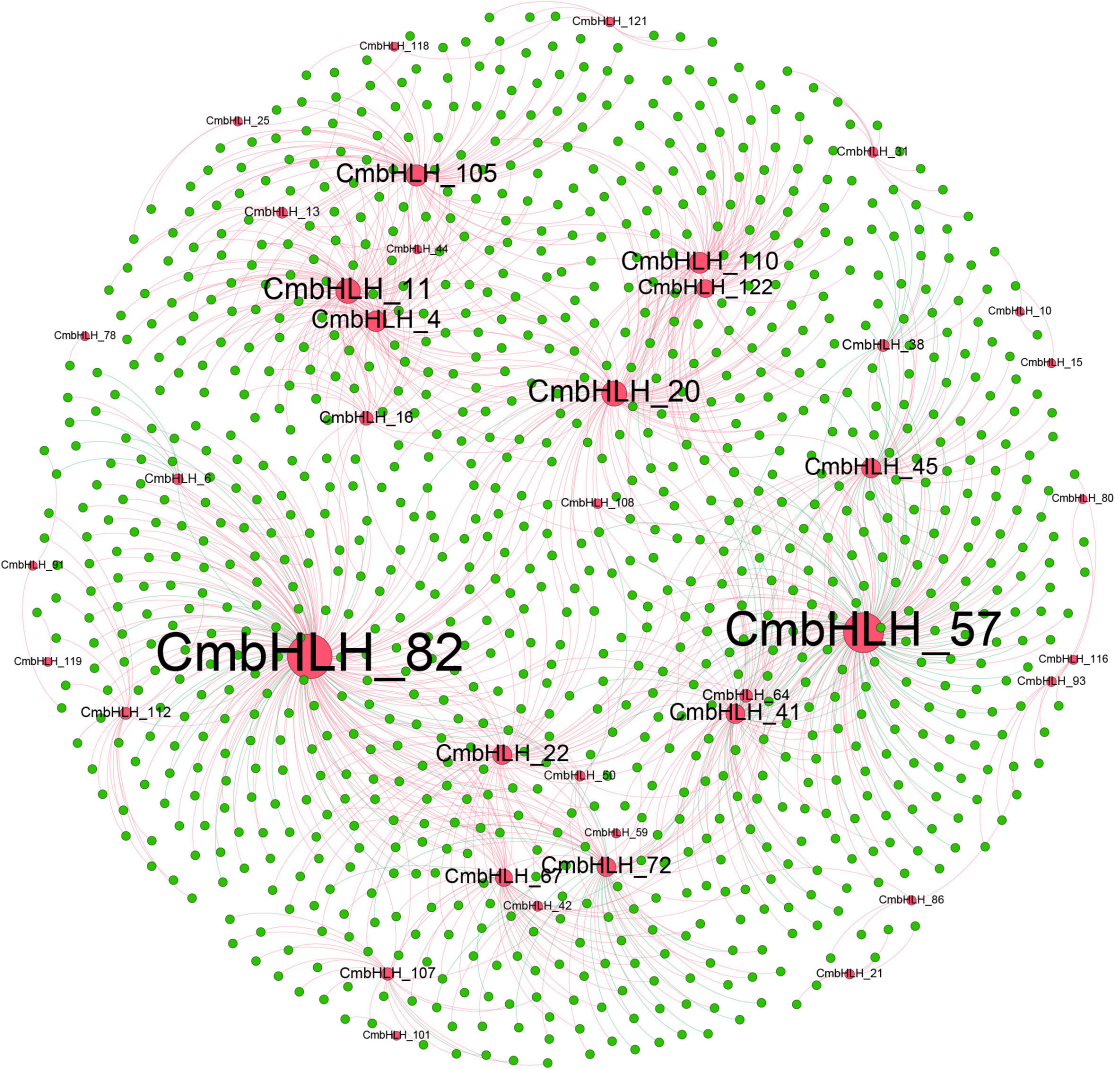
Co-expression networks of TFs and other genes. Red nodes are bHLH, green nodes are other genes, red lines indicate positive correlations, green lines indicate negative correlations. Shown the gene relationships with correlations >0.8 and p<0.05.

A co-expression network was constructed based on the correlation of expression levels of *bHLH* and other genes during fruit development. The network contained 1045 nodes (96.17% of other genes and 3.83% of *bHLH* transcription factors) and 1272 edges (87.74% of pairs were positive correlated and 12.26% were negative correlated) (**Figure 8**). In general, we consider the expression of transcription factors and target genes to be positively correlated. The two most highly connected transcription factor are *CmbHLH82* and *CmbHLH57*, which are positively correlated with the vast majority of other genes. From the expression patterns of *CmbHLH*82 and *CmbHLH*57 in YF1, YRZ and YM1, it can be seen that their expression decreases with the increase of development, which indicates that transcription factors are crucial in the gene initiation stage.

### Expression patterns of *CmbHLHs* in response to dryocosmus kuriphilus yasumatsu infestation

**Figure 9:**
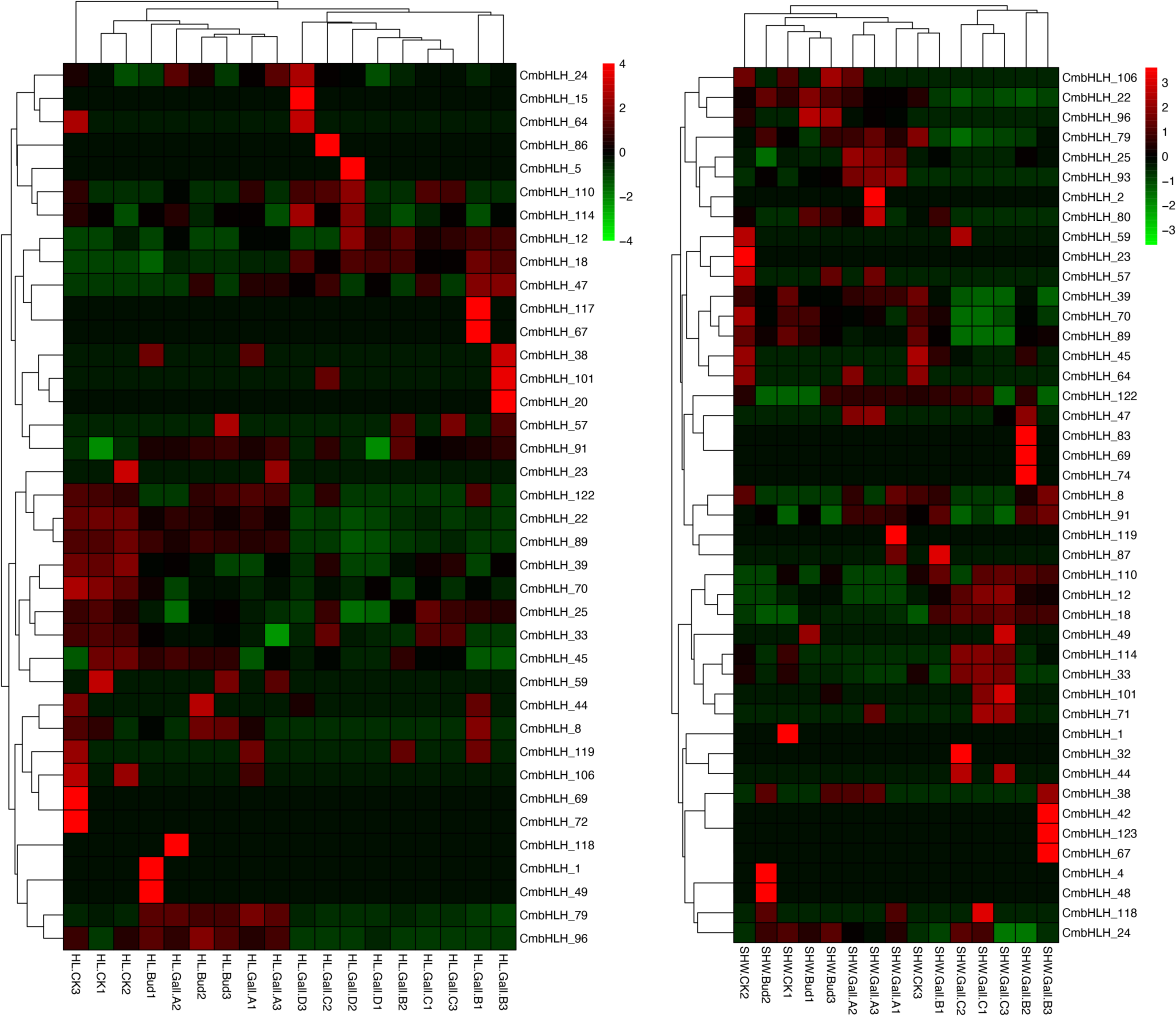
Two different varieties of chestnut (HL, SHW) in response to D. kuriphilus infection of *CmbHLH* staged expression map. In general, dryocosmus kuriphilus yasumatsu infection greatly disrupted the expression pattern of *CmbHLH* in different organs of chestnut, and also activated some *CmbHLH* expression. This may indicate that *CmbHLH* also plays an important role in coping with biological stress.

In order to further analyze the function of *CmbHLHs* genes in resistance to *D. kuriphilus* infestation, RNA-seq data of *CmbHLHs* genes from our previous studies were used to investigate the dynamic changes during four leaf gall-formation stages caused by *D. kuriphilus*. We identified 15 *CmbHLHs* genes that were progressively less expressed with *D. kuriphilus* infestation. These genes included *CmbHLH164*, *CmbHLH42*, *CmbHLH163*, *CmbHLH124a*, *CmbHLH96a*, *CmbHLH60*. They continued to be down-regulated in expression under the influence of *D. kuriphilus* infestation. At the same time, 21 *CmbHLHs* genes showed an up-regulated trend in expression with the infested of *D. kuriphilus*. These genes include *CmbHLH63*, *CmbHLH51*. Overall, most *CmbHLHs* genes showed down-regulated expression when infested by *D. kuriphilus*. The persistent down-regulation of *CmbHLH42* may indicate that D. kuriphilus affects downstream anthocyanin biosynthesis by affecting the expression of *CmbHLH4*2. In addition, genes such as *CmbHLH63* and *CmbHLH51* showed up-regulated expression when chestnut leaves are infected by *D. kuriphilus*. This may indicate that CmbHLH63 and CmbHLH51 may play an important role in the process of chestnut resistance to biological stress. The down-regulated expression of these genes may be closely related to the formation of galls in chestnut leaves when they are invaded by *D. kuriphilus* (**Figure 9**).

**Figure 10:**
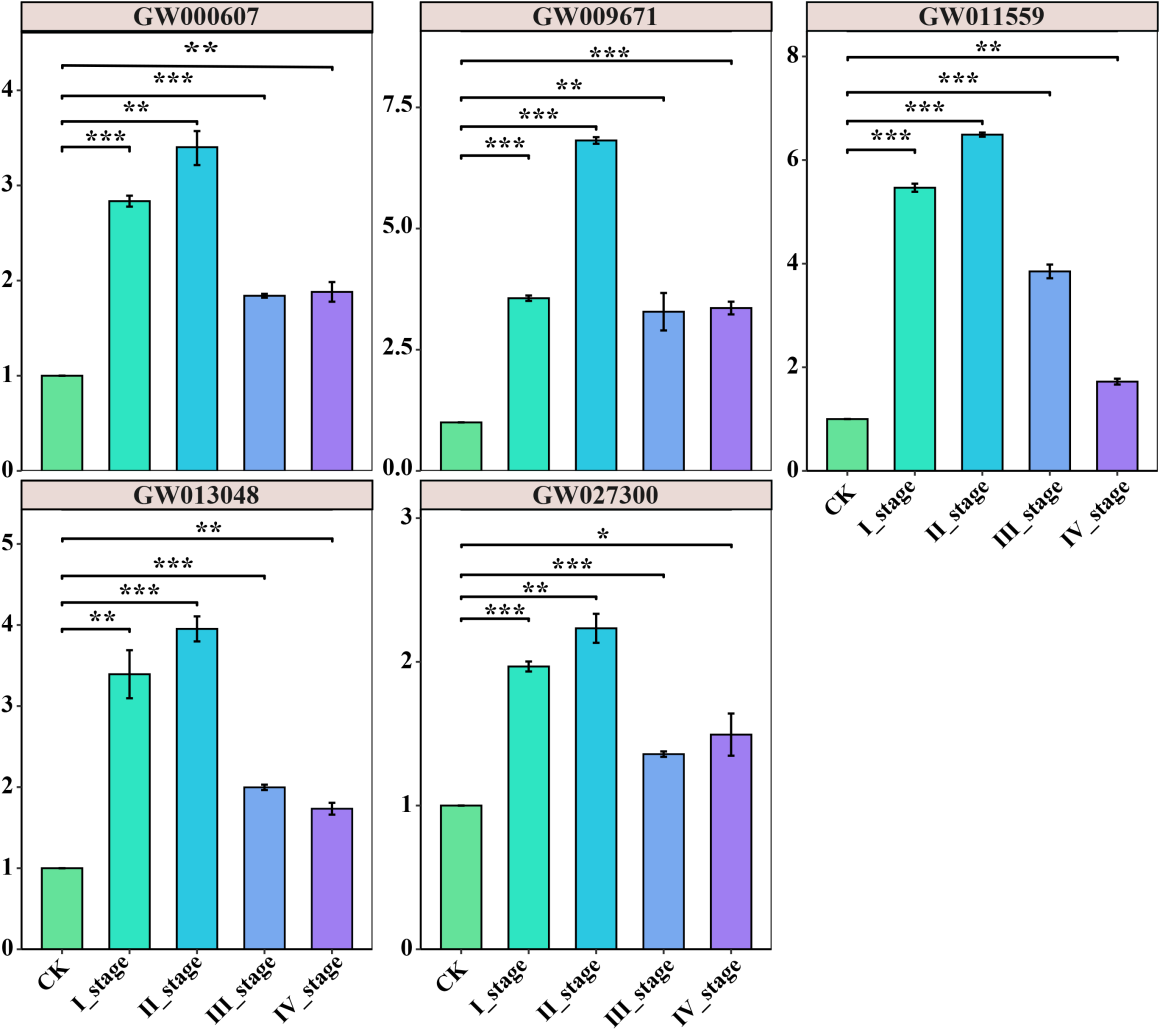
qRT-PCR validation of five genes from eight subfamilies throughout the four stages of chestnut seed development. p < 0.05 (*); p < 0.01 (**); p < 0.001 (***).

To further evaluate the potential functionality of these genes, five genes from eight subfamilies were selected and performed qRT-PCR analysis to examine the expression patterns of these representative genes throughout the four stages of chestnut seed development (**Figure 10**). The *CmbHLH* genes showed different expression patterns at different stages of seed development, suggesting its diverse regulatory roles. All genes were expressed in different stages, and five genes were significantly up regulated at I_stage and peaked at II_stage. Subsequently, during the III_stage and IV_stage, there was a declining trend in gene expression levels. However, they remained significantly higher than the control level. These five representative genes play a pivotal role in seed development (**Figure 10**).

## Discussion

The bHLH gene family has been extensively characterized in various plant species; however, systematic studies in chestnut remain lacking. Compared with other species, chestnut genome research is still at an early stage. In this study, 124 bHLH genes were identified from the chestnut genome and classified into eight subgroups. Among them, 18 CmbHLH members harboring motif 3 also possess an additional highly conserved seven-amino-acid domain, which may indicate functional conservation. The total number of bHLH genes identified in chestnut is lower than that in Arabidopsis and maize. Phylogenetic analysis of CmbHLH, AtbHLH, and ZmbHLH proteins revealed a mixed clustering pattern, suggesting that the bHLH gene family predates the divergence of these lineages and subsequently underwent species-specific expansion and diversification. Furthermore, the co-expression network analysis revealed that several CmbHLH genes exhibit strong correlations with genes involved in stress responses, hormone signaling, and secondary metabolism, implying their potential regulatory roles in diverse biological processes and adaptation mechanisms. These findings lay a foundation for future functional characterization of bHLH transcription factors in chestnut.

RNA-seq analysis revealed that a substantial number of CmbHLH genes are transcriptionally active during chestnut fruit development, suggesting their potential regulatory roles in this biological process. Notably, CmbHLH124 exhibited high expression during the early stage of fruit development, followed by a gradual decline as the fruit matured. Similarly, CmbHLH4, a homolog of ICE1, displayed a comparable expression pattern. ICE1 has been reported to regulate lateral bud growth in Arabidopsis (Du et al., 2023), indicating that its chestnut ortholog may play a comparable role in early developmental regulation. CmbHLH8 and CmbHLH86, homologous to LRL2 and PIF3 respectively, also showed high expression in the early developmental stages. PIF3 is a well-characterized regulator in *Arabidopsis*, known to mediate ethylene-induced hypocotyl elongation by promoting microtubule reorganization (Yin et al., 2023). The elevated expression of CmbHLH86 during early fruit development may be attributable to post-anthesis ethylene signaling, with its expression declining as fruit matures. This trend was also observed in other genes such as CmbHLH96 and CmbHLH104, suggesting that these transcription factors may collectively function during the initial phases of fruit set and early growth (Kuang et al., 2023; Gao et al., 2023). Conversely, CmbHLH16, the chestnut homolog of UNE10, exhibited an increasing expression trend throughout fruit maturation, implying its involvement in the later stages of fruit development. A similar pattern was observed for CmbHLH6, which showed progressive upregulation during the ripening process. In rice, the orthologous gene OsbHLH6 has been shown to mediate responses to jasmonic acid (JA) and to contribute to biotic and abiotic stress susceptibility (Meng et al., 2020). Therefore, it is plausible that the expression of CmbHLH6 in chestnut is induced by endogenous JA signaling, facilitating its functional participation in the ripening program.

Finally, we screened five genes for qRT-PCR analysis and functional trait validation. The results revealed significant up-regulation of these genes during the four stages of leaf gall formation, indicating their potential responsiveness to stress across various stages. These differential expression patterns underscore the temporal specificity and functional diversity of bHLH transcription factors in chestnut fruit development and maturation. Further functional validation is necessary to elucidate their precise regulatory mechanisms.

## Conclusions

In this study, the bHLH gene family of Chinese chestnut was described at the genome level. Their gene structure, chromosomal distribution, phylogenetic relationship, and various expression patterns are proposed. We discussed the evolutionary relationships, expression and function of *bHLH*s in detail. These results provided a solid basis for further studies on the biological functions and evolution of chestnut bHLH. Our analysis will boost future functional analysis of bHLHs in chestnut and contribute to molecular breeding for improving yield, stress tolerance and grain quality.

## Data Availability

Publicly available datasets were analyzed in this study. This data can be found here: All data generated or analysed during this study are were obtained from publicly available databases. The reference genome of Chinese chestnut was downloaded from Hardwood Genomics Project(HWG, https://www.hardwoodgenomics.org/Genome-assembly/1962958). The raw sequencing data of transcriptome data obtained through the accession number PRJNA574282 and PRJNA512447 from the SRA database (https://www.ncbi.nlm.nih.gov/sra).
