# Supplementary material for: Genome-wide analysis of bHLH transcription factor family reveals their involvement in kernel development and biotic stress responses in Chinese chestnut
Source: Front Plant Sci. 2025 Sep 18;16:1627760. doi: 10.3389/fpls.2025.1627760 (PMC12488670; doi:10.3389/fpls.2025.1627760)
Supplement: Supplementary file 1 [file Supplementaryfile1.docx]

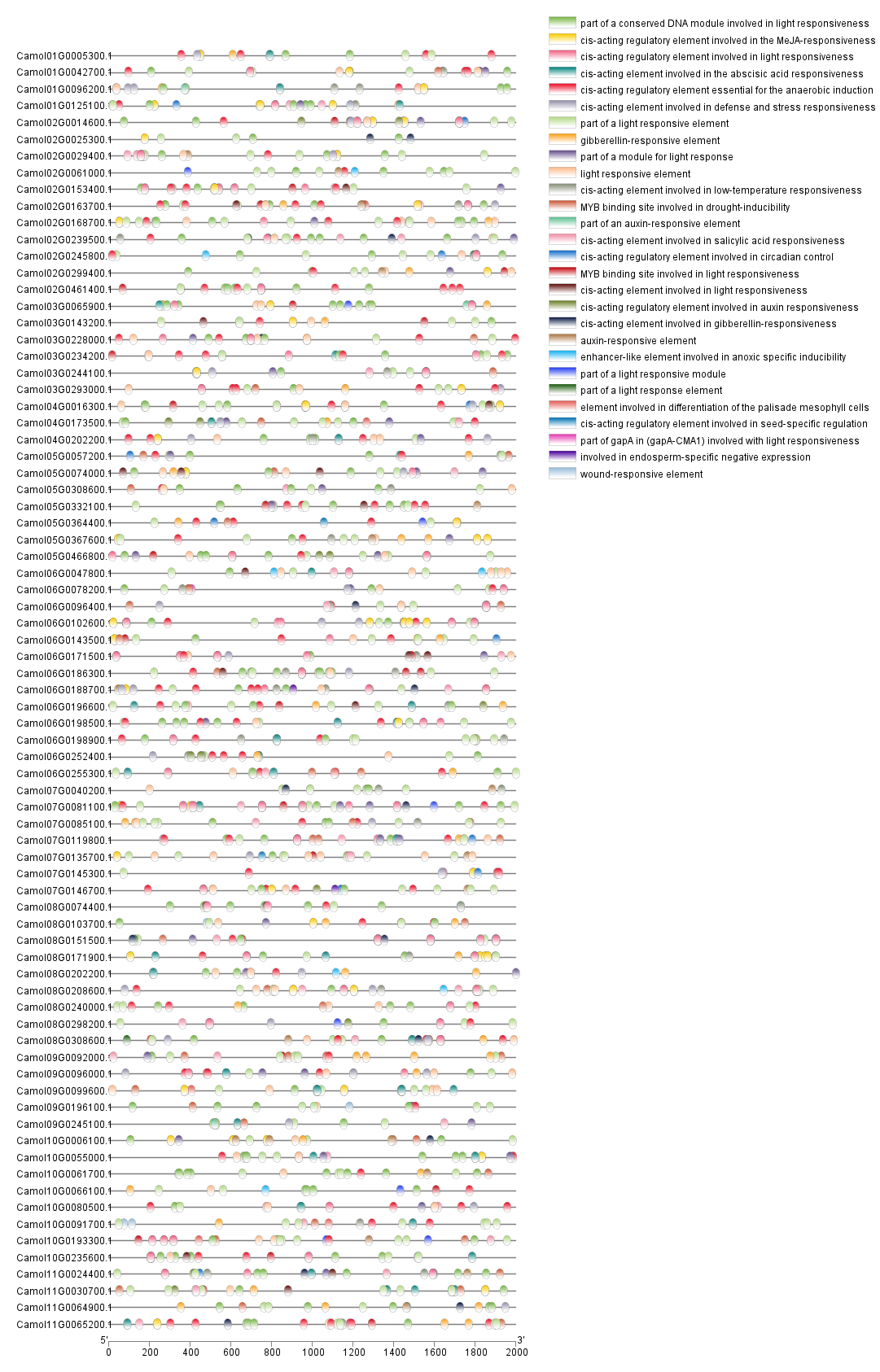


Supplementary Fig 1: Promoter element analysis of CmbHLH transcription factor family of *Castanea mollissima.*
